# An intelligent agent for sentence completion test: creation and application in depression assessment

**DOI:** 10.3389/fpsyg.2025.1649905

**Published:** 2025-08-12

**Authors:** Yuchen Huang, Mengxiao Lei, Hanyu Zhang, Lu Zong, Bin Zhu, Hong Luo

**Affiliations:** ^1^Affiliated Mental Health Center and Hangzhou Seventh People’s Hospital, Zhejiang University School of Medicine, Hangzhou, China; ^2^Hangzhou PsychSnail Technology Company Limited, Hangzhou, China; ^3^Hangzhou Yunqi Interdisciplinary Technology Research Institute, Hangzhou, China; ^4^Suzhou Guangzhinian Technology Company Limited, Suzhou, China; ^5^School of Journalism & Communication, Hangzhou City University, Hangzhou, China

**Keywords:** sentence completion test, projective test, large language models, AI agent, depression assessment

## Abstract

During large-scale psychological screening, traditional self-report questionnaires face challenges like response deception or social desirability bias, while the Sentence Completion Test (SCT) as a projective technique shows potential but is limited by manual scoring and high costs. Leveraging advancements in Large Language Models (LLMs), this study integrates SCT’s theoretical framework with LLM capabilities to develop a specialized set of SCT items for depression assessment in Chinese university students, using a self-built intelligent agent across three progressive empirical studies. Results show the agent demonstrates good reliability (Cronbach’s *α* = 0.89–0.92) and validity, with high consistency to manual scoring (*r* = 0.96), significant criterion correlations with the Beck Depression Inventory (*r* = 0.89) and Self-Rating Depression Scale (*r* = 0.85), confirmed structural validity via exploratory factor analysis. Furthermore, the intelligent agent could identify most invalid responses (F1 = 0.94, Accuracy = 0.99, Precision = 0.99, Recall = 0.90). This research marks a key milestone in SCT’s intelligent transformation, driving innovation in psychological assessment and offering new academic and practical pathways.

## 1 Introduction

Psychological assessment is an essential component of mental health service systems. How to conduct efficient and accurate psychological assessment is a prerequisite for providing effective interventions (Jiang et al., 2022). College students are a high-risk group for depressive symptoms, with detection rates showing an upward trend (Chen et al., 2022), making timely depression measurement crucial (Guo et al., 2024). In psychological health screenings, questionnaire methods are commonly used for assessment (Li, 2000). While questionnaires have advantages such as simplicity and high standardization, respondents can easily fake responses or be influenced by social desirability (Podsakoff and Organ, 1986). Additionally, due to limited response options, questionnaires may struggle to measure traits beyond available options and may not reflect respondents’ true psychological characteristics (Huang, 2016). From the perspective of assessment efficiency and accuracy, projective techniques can play a more important role (Huang, 2016; Ares and Varela, 2018; Tong, 2002).

Projective tests, as unstructured stimulus assessment tools, are theoretically based on the assumption that individuals project their internal psychological traits in ambiguous situations (Lindzey, 1952). Compared to questionnaire tests, their advantages lie in bypassing defensive mechanisms (Rabin, 1986), obtaining more accurate natural responses (Dumitrescu, 2023), and increasing engagement (Ares and Varela, 2018). Classic projective techniques include the Rorschach Ink Blot Test, Thematic Apperception Test, House-Tree-Person Test, and Sentence Completion Test (SCT). Among these, Thematic Apperception Test and RIBT are typically administered individually in clinical counseling and assessment, and their interpretation process is rather cumbersome, requiring professional training for effective application (Huang, 2016). The test formats of House-Tree-Person Test, Thematic Apperception Test, and Rorschach Ink Blot Test determine that the psychological traits they can assess are limited (Tong, 2002). SCT, due to its feasibility for group administration, low professional threshold, ease of objective scoring, and high application flexibility (Huang, 2016), is considered the projective tool with the greatest methodological advantages (Murstein, 1965; Sherry et al., 2004). This technique captures potential psychological characteristics through a series of sentence completion tasks (Holaday et al., 2000). Despite controversies, its reliability and validity have been verified by many studies (Bronlet, 2025; Huang, 2016; Sherry et al., 2004), and it is widely popular in clinical, child, and school psychology fields (Holaday et al., 2000; Piotrowski 2018; Ridgway et al., 2024). However, traditional manual scoring has limitations such as dependence on expert experience (Piotrowski, 2018) and time-consuming complexity (Huang, 2016), with even experienced raters needing 20 min to complete an assessment (Hy and Loevinger, 1996). Artificial intelligence technology provides a solution to address such limitations.

Large Language Models (LLMs), designed specifically for language generation and other natural language processing (NLP) tasks (Demszky et al., 2023), can achieve deep parsing of text semantics (Markowitz, 2024; Kuzmin et al., 2024) and efficiently process large-scale complex texts (Demszky et al., 2023). NLP not only demonstrates excellent capabilities in text emotion recognition (Markowitz, 2024; Rathje et al., 2024; Kuzmin et al., 2024) but also shows stronger stability than manual scoring in some text evaluation tasks (Xu et al., 2024), which helps mitigate subjective fluctuations in manual scoring (such as fatigue and mood) (Davier et al., 2023). These technical advantages have demonstrated extensive application potential in mental health assessment scenarios: For example, LLM can convert unstructured clinical interviews into structured questionnaires such as Patient Health Questionnaire-8, predict the severity of depression by simulating patients’ response patterns, and significantly improve diagnostic accuracy (Rosenman et al., 2024). In the task of symptom definition, it can accurately identify key segments related to depression in conversations (such as stressors and emotional states) and generate clinical summaries to assist doctors in decision-making (So et al., 2024). Meanwhile, the PsychoGAT framework uses LLM to transform traditional scales into interactive novel formats, infer psychological characteristics through players’ behavioral data, and effectively reduce resistance to testing (Zhang et al., 2024).

In the concept of combining LLM with projective tests, since language is the core of all psychological fields (Demszky et al., 2023), and the core architecture of LLMs revolves around language understanding and generation tasks (Rathje et al., 2024), SCT has greater data interpretability (Sherry et al., 2004) and potential to leverage NLP technology advantages compared to other projective methods that rely on ink blot or image interpretation. Intelligent Agent systems, by standardizing human-machine interaction, can solve the output instability problem of LLMs caused by prompt input variations (Park et al., 2024). The component workflow integrated by agents can control the evaluation process in stages (Yang et al., 2024; Zhang et al., 2024), avoiding logical gaps and performing real-time verification (such as response time thresholds, invalid response filtering) to ensure data quality (Xi et al., 2025). This technology integration solution preserves the ecological validity of projective tests, enhances the application advantages of SCT as a language carrier in NLP technology (Demszky et al., 2023), reduces the scoring cost of traditional methods through LLMs (Bronlet, 2025), and standardizes human-machine interaction through intelligent agents (Xi et al., 2025), providing technical support for large-scale psychological screening with SCT.

However, how to implement the combination of SCT and intelligent agents, to our knowledge, has not yet been clearly researched or systematically evaluated. Bronlet (2025) used highly validated SCT as an indicator of behavioral variables and applied LLM for text data classification, but did not conduct interference sample detection, had a small sample size (*N* = 58), and direct application of LLM for text classification has limitations such as low reproducibility of data analysis and insufficient standardization (Markowitz, 2024). Given the aforementioned research gaps and limitations of existing methods, this study focuses on the context of depression assessment among Chinese college students, developing an agent based on the principles of SCT and conducting a series of empirical studies. By integrating SCT principles with the advantages of intelligent agents, we hope to build an efficient, standardized interactive, and widely adaptable assessment method.

## 2 Materials and methods

Although there is currently no research paradigm for LLM agents using SCT principles, and Chinese scholars have not yet developed an SCT to measure the depression levels of Chinese college students, relevant methods can still be borrowed from existing studies. First, the item development and scoring criteria for the SCT can refer to the design processes of other related research. Second, the reliability and validity of the agent’s scoring can be verified through manual scoring quantification. Third, the text processing performance of the agent can be evaluated using paradigms from other machine learning text classification studies. Through the design of three experiments, this research expects to validate the following in each experiment: (1) The independently developed SCT items exhibit good reliability and validity; (2) The LLM-based agent can be applied to the scoring of depression levels, with high reliability and validity in its scoring, showing a strong positive correlation with manual scoring; (3) The agent can effectively identify invalid responses and process invalid data accordingly.

### 2.1 Experiment 1: development and preliminary reliability & validity test of SCT items

The purpose of Experiment 1 is to compile SCT items for measuring the depressive psychology of Chinese college students based on the SCT compilation principles, and to preliminarily test the reliability and validity of the compiled SCT items.

#### 2.1.1 Item design and screening

When designing SCT items, considerations include target population, clinical orientation, and construct alignment (Rogers et al., 2003). Regarding the target population, since SCT is widely applied in clinical, child, and school psychology fields (Huang and Zheng, 2000; Li, 2000; Ridgway et al., 2024), combined with the fact that college students are a high-risk group for depressive symptoms (Chen et al., 2022), this study takes the level of depression among college students as its research content. Based on the length of the sentence stems, items in the SCT can be categorized into two types: high-structured and low-structured (Huang, 2016). Examples of low-structured items include “I think…” and “I feel…,” while high-structured items are exemplified by “The body part I am most satisfied with is…” and “The subject I hate the most is….” Barton et al. (2005) advocated for low-structured designs, as the ambiguous sentence structure is more likely to elicit unrestrained spontaneous responses from respondents, whereas other research indicates that high-structured items can reduce semantic ambiguity (Trites, 1956) and strengthen the validity of affective projection (Turnbow and Dana, 1981). Huang (2016) incorporated both structures when designing the Chinese version of the SCT, and this study follows suit.

When designing items, the choice of personal pronouns also needs to be considered (Huang, 2016). Drawing on evidence that individuals with depression exhibit high self-focus and a tendency to use first-person pronouns in communication (Lyu et al., 2023; Rude et al., 2004), we primarily employed first-person pronouns in constructing the stems. For the thematic design of the items, all depressive themes mentioned in the reviewed materials (31 in total) were summarized by reviewing literature (Huang, 2016; Zhang et al., 2024) and established scales (Symptom Checklist-90, Self-Rating Depression Scale, and Beck Depression Inventory), with 4–5 stems designed for each theme.

A focus group consisting of 4 researchers from relevant disciplines was formed. Members completed item optimization through multiple rounds of discussions. The first round prioritized screening items with cultural adaptability and cognitive characteristics of Chinese college students. The second round eliminated items with limited test validity, such as “The cafeteria food…” “My weight…” and other themes reflecting specific situations, ultimately retaining 41 items.

In the pre-study phase (*N* = 16), based on participant feedback, items were further evaluated and screened. Stems triggering invalid responses were eliminated, such as “After graduation…” which easily produced career planning-type answers; stems with insufficient discriminative validity were excluded, such as “Thinking of sad things…” which showed high responses in non-depressed groups. After analysis, the total number of items was determined to be 17. To ensure items had expert content validity, 3 experts with SCT application experience were invited to review each item, meeting the standard for expert review (Shi et al., 2012). Following Huang (2016)'s review principles, items were categorized as follows: (1) Retain original item. If all three experts approved the item as suitable, it was retained without modification. (2) Modify item. If one or more experts considered the item suitable after modification, the original item was modified according to expert opinions. (3) Delete original item. If one or more experts considered the item unsuitable, it was deleted without modification. After expert review: 16 items were approved and retained; “My sleep quality…” was changed to “My sleep situation…” to increase information richness. After other two participants confirmed that the modified items had clear meaning and testing feasibility, the formal item content was finalized (Huang, 2016).

#### 2.1.2 Participants and data collection

The target sample size for criterion-related validity analysis was determined using G*Power software (Faul et al., 2009). Calculations indicated that to satisfy the requirements of a medium effect size (*ρ* = 0.30) and statistical power (power = 0.8) for correlation analysis, a minimum sample of 82 participants was needed. To ensure sample diversity and support precise college student sampling, college student participants (aged 18–22) were recruited through Wenjuanxing sample service, covering 15 provincial regions in China. All participants participated voluntarily, signed informed consent forms, and received compensation from the platform upon completing the questionnaires. A total of 103 valid samples containing SCT and criterion-related questionnaires were collected (with an average actual response time of 216 s). Two responses were excluded due to inconsistencies in measurements of the same topic between the SCT and the criterion-related questionnaire—for example, stating “My sleep situation is very good” in the SCT while selecting “My sleep is very poor” in another questionnaire. After excluding 2 questionnaires with contradictory answers, 101 valid samples remained, meeting the sample size requirements for criterion-related validity. The questionnaire administration sequence was reasonably arranged, requiring participants to complete the SCT questionnaire first, then the criterion-related questionnaires.

The sample for test–retest reliability analysis was obtained through follow-up 1 week later. To save resources, 40 individuals were followed up for a second time, and the sample size falls within the widely accepted range (Guo, 2010; Huang, 2016). One response was excluded as it was blank and could not be analyzed, leaving 39 valid samples.

#### 2.1.3 Scoring criteria

Because commonly used depression scales such as the PHQ-9, BDI, and SDS all use a 4-point scale in their scoring, we therefore also use 4-point to measure the degree of depression reflected in the sentences: 0 represents neutral or positive emotions, and the score increases as the severity of depression increases, with a separate category for invalid responses.

Studies typically define six categories of defensive responses in SCT (Loevinger and Wessler, 1970; Ridgway et al., 2024): omission, denial, redundancy, flippancy, comments about the test, and simple association. When applying psychological measurement tools to new cultural contexts, directly copying the language, concepts, and scoring criteria of the original measurement tools is often insufficient; it is necessary to examine and adjust aspects such as semantics and cultural relevance (Hambleton et al., 2005). Furthermore, a more concise and clearer scoring classification system is of great significance for improving the efficiency and quality of measurement tools in practical applications (Suzuki and Ponterotto, 2008).

Members of the focus group constructed 10 sets of 160 Chinese invalid responses based on the items, which were then discussed and categorized. After preliminary coding, the six types were further integrated into four categories (Table 1):

1. Content Omission. Includes the original “omission” type. “Omission” manifests as missing key information, making it impossible to effectively assess depression status. We list it as a separate category.
2. Avoidance Interference. Integrates the original “denial” and “comments about the test” types. “Denial” expresses defensive reactions by negating the stem, while “comments about the test” shifts focus to opinions about the test itself, deviating from the core content of depression assessment. Both cases essentially avoid the core issues of assessment in different ways, interfering with obtaining accurate depression state information.
3. Meaningless. Encompasses the original “redundant response” type. Such responses have no substantial content and cannot effectively judge depression status, emphasizing the lack of content value in these responses.
4. Off-topic. Combines the original “flippant response” and “simple association” types. “Flippant response” provides unrealistic answers without serious consideration of depression-related issues; “simple association” provides answers unrelated to the question, deviating from the theme of depression assessment. Both types fail to provide effective information about depression assessment, only deviating from the theme from different angles.

**Table 1:** Definition and examples of four types of invalid responses.

Type	Components	Typical example	Behavioral characteristics
Content omission	Omission	My mood is often (blank)	Information completeness deficiency
Avoidance interference	Denial + Test Comments	When I make decisions, I do not make decisions	Cognitive avoidance tendency
Meaningless	Redundant Response	Looking back, I feel funny	Content validity deficiency
Off-topic	Flippant Response + Simple Association	Facing the future, lying flat	Lack of task compliance

Human scoring: Two graduate students in psychology were selected. Before scoring, they standardized evaluation criteria, negotiated principles for handling divergent responses, etc. Each person completed one full scoring, with discussions and different opinions permitted during the process. After all scoring was completed, an expert reviewed the manual scoring data and made annotations.

### 2.2 Experiment 2: development and reliability & validity test of the SCT intelligent agent

The purpose of Experiment 2 is to evaluate the data analysis performance of an LLM-based agent in interpreting SCT results, with a focus on verifying its reliability and validity.

#### 2.2.1 Agent development

The Coze platform was chosen for the development and design of the intelligent agent. This study chose the single Agent (LLM mode), simplifying task logic while supplementing with workflow modules to achieve conversation constraints and establish clear interaction processes. For LLM selection, considering that Doubao Universal Pro received the highest score for Chinese language capability in the subjective evaluation of LLMs by Zhiyuan Research Institute in December 2024, and with an accurate understanding of Chinese language contexts (Economic Observer, 2024), we conducted a small-scale assessment of Doubao Universal Pro’s SCT discrimination capability. In 20 SCT data samples, this LLM’s identification rate based on prompts was 96.56%, meeting experimental expectations.

**Figure 1:**
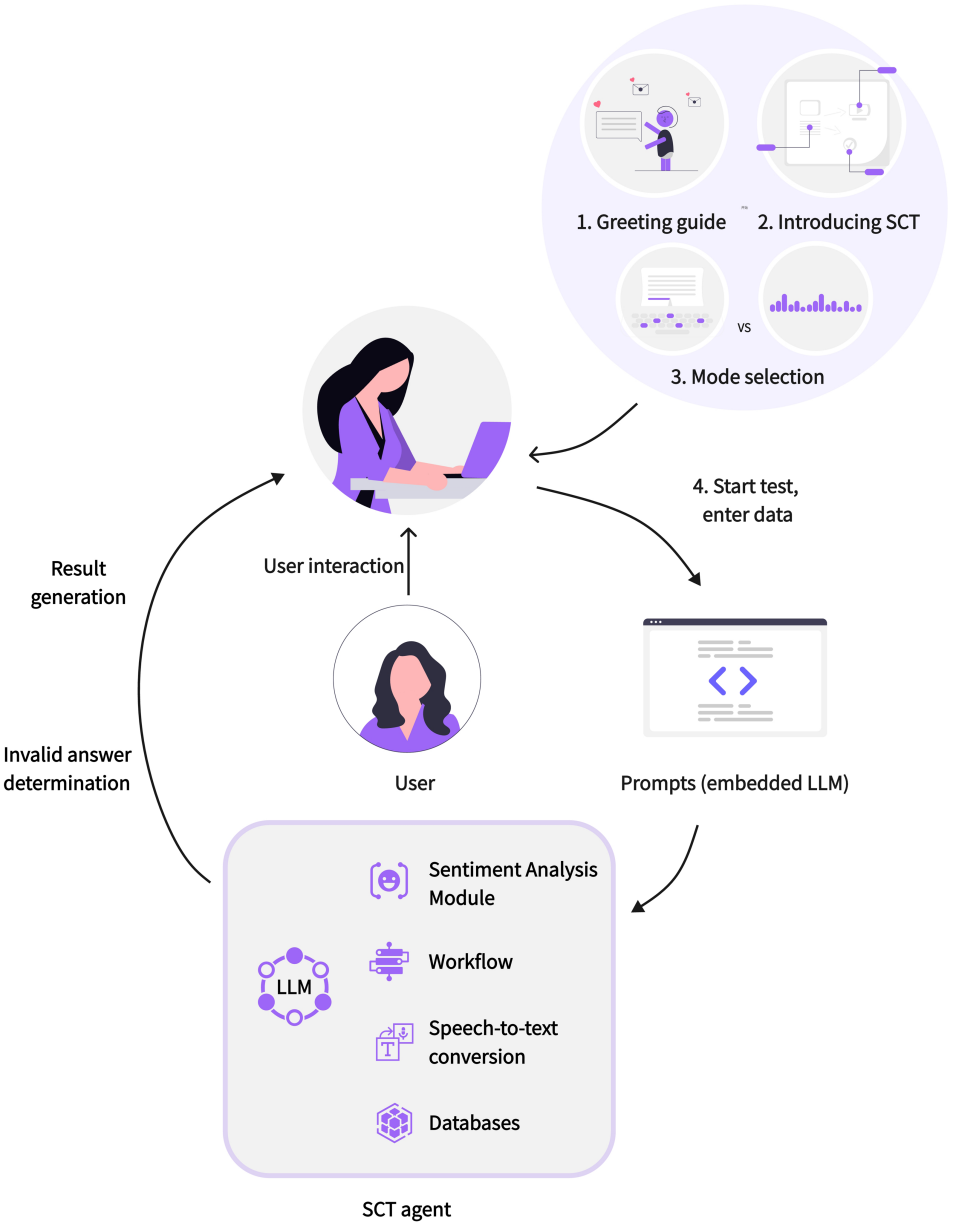
The agent architecture.

**Figure 2:**
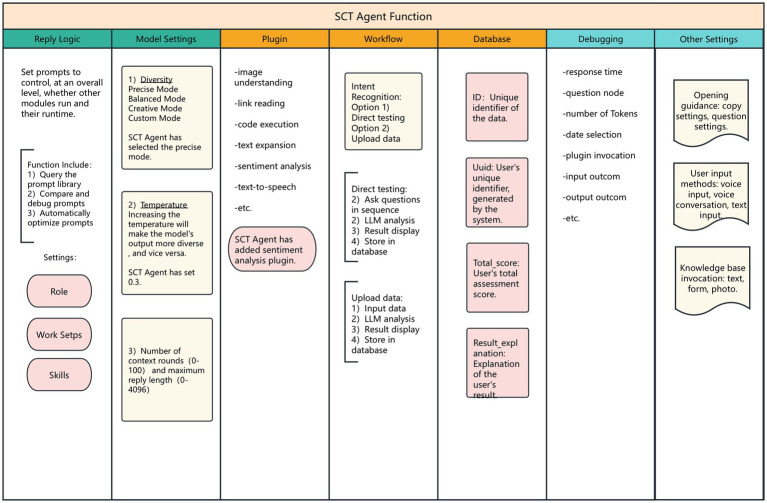
The functional modules of the SCT agent.

In intelligent agent development, emphasis was placed on using workflows for standardized interaction. A workflow refers to a functional component that enables the intelligent agent to automatically execute a series of tasks in a specific order and according to rules, with the core being nodes. Each node is an independent component with specific functions, such as code nodes, SQL custom nodes, plugin nodes, Q&A nodes, loop nodes, etc., representing an independent step or logic. These nodes are responsible for processing data, executing tasks, and running algorithms, and they typically all have input and output parameters. By referencing output, nodes can be connected to form a seamless operation chain, supporting various variable types including String, Integer, Number, Boolean, Object, File, and Array. This intelligent agent includes intent recognition, code writing, LLM calling, data input, text output, database addition, and other nodes. The Agent Architecture is shown in Figure 1. The functional modules of the SCT agent are in Figure 2.

**Figure 3:**
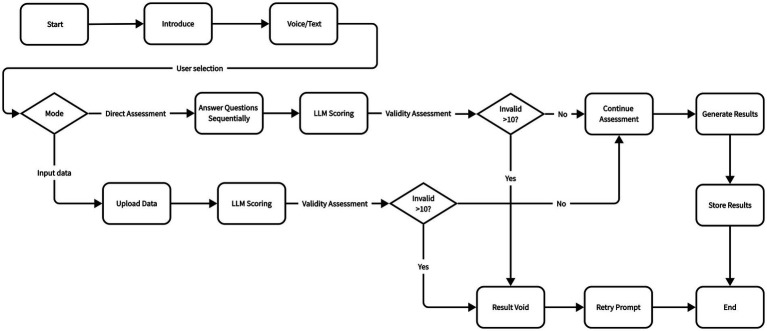
SCT flowchart.

The entire intelligent agent workflow is divided into four parts, as shown in Figure 3. In the first phase, opening statements and prompts are designed to guide users in initial interaction, introducing SCT test principles and providing examples. Both voice calls and text responses are offered. Users are informed they can choose direct assessment or input data analysis. In the second phase, intent recognition serves as the starting node for calling the workflow. When users reply “direct assessment,” the workflow offering corresponding SCT items in sequence. When users reply “input data,” the workflow enters data input analysis mode. In the third phase, formal assessment begins. If “direct assessment” is chosen, after finishing the SCT items, the LLM starts to evaluate depression levels for all responses. If responses contain the 4 defensive reactions mentioned above, they will be identified by LLM as invalid. If “input data” is chosen, a dialog box is provided for text data input, calling the LLM for depression level assessment, with invalid response identification as above. In the fourth phase, results are generated. Based on LLM judgment results, each user response is evaluated on a 0–3 point scale or as an invalid response with corresponding justification, finally generating result interpretation and storing in the database.

Noergaard et al. (2018) established an exclusion criterion for invalid questionnaires, whereby responses with a missing rate exceeding 2/3 in their survey were excluded. With reference to this criterion, 10 invalid responses are set as the judgment threshold. If total invalid responses exceed 10, the result is considered invalid, no score is output, and users are prompted to respond again.

#### 2.2.2 Participants and measures

To validate the correlation between the scores assigned by the SCT agent and manual scores, we input the 101 SCT result datasets collected in Experiment 1 into the agent for re-scoring and compared the results with manual scores.

To explore the model structure of the scoring result by the SCT agent, we conducted an exploratory factor analysis. We recruited 261 Chinese college students across regions (aged 18–22) through the Wenjuanxing platform, completing both SCT and BDI (This sample is also used for criterion-related analysis). The SCT questionnaire was the same as in Experiment 1, removing the “If I were to score myself” item, for a total of 16 questions. During data processing, 8 invalid responses were excluded. Sample size estimation was based on the formula as follows:


$$N=\frac{Z^{2}\times P\times (1-P)}{E^{2}}$$

In this study, *Z* = 1.96 (i.e., confidence level of 95%), *E* = 5%, *p* = 20.8%. According to meta-analysis, the detection rate of depressive symptoms among Chinese college students is 20.8% (Chen et al., 2022), calculating that this study required a sample size of at least 253 people, meeting sample requirements.

In the criterion-related analysis, Sample 1, which is the same as the sample used for the exploratory factor analysis, was employed to analyze the correlation between the SCT and the BDI. Sample 2 was used to analyze the correlation between the SCT and the Self-rating Depression Scale (SDS). To cover diverse sources and enhance the robustness of the results, 118 college student participants (aged 18–22) were recruited through the Credamo platform (Similar to Wenjuanxing, is one of the commonly used data collection platforms in China.). Six responses were excluded for failing the attention check questions (For instance, when an attention check question required selecting option C, the participants chose A instead), resulting in 112 valid samples. The target number of samples for criterion-related validity analysis was determined using G*Power (Faul et al., 2009). To meet the requirements of medium effect size *ρ* = 0.30 and power = 0.8 for correlation analysis, a minimum of 82 participants was needed. Both criterion-related samples in this experiment satisfied this requirement (*N* = 253; *N* = 112).

### 2.3 Experiment 3: evaluation of invalid response recognition ability

The purpose of Experiment 3 is to evaluate the processing capability of this agent in the data screening stage, with a focus on verifying its performance in the classification task of invalid responses.

#### 2.3.1 Pilot test and participants

The samples containing 20 invalid data points collected in Experiment 2 were input into the intelligent agent for judgment. Of the 20 invalid responses, 18 incomplete responses consisting of punctuation marks like “.” and “,” could all be identified as content omission type, and 2 unclear words “If I make a mistake, I would beef” and “My heartbeat goes boom boom boom!” could be identified as meaningless type, with no scoring for these 20 invalid responses. This test verified the intelligent agent’s good data screening capability in complex data environments.

Sixty college students were recruited through the Credamo platform, required to complete 16 SCT items, with 8 items chosen for giving invalid responses, marked with (invalid) at the end of response. The invalid responses were not defined for participants to collect open-ended data. The remaining 8 items required serious completion. After collection, invalid responses were coded and classified, with data for each classification appearing multiple times in existing 4 types, reaching sample saturation and meeting qualitative research sample size requirements (Xie and Chen, 2021).

#### 2.3.2 Evaluation metrics

During the collecting process, it was found that the invalid responses marked by participants included the four types of defensive reactions preset earlier, reaching thematic classification saturation (Xie and Chen, 2021). Another finding was disguised invalid responses, i.e., semantically clear, not deviating from the measurement theme, but marked as invalid by participants, such as “My mood is often very excited (invalid)” and “If I make a mistake, I would regret it (invalid).” Such responses are, in fact, a form of falsification that cannot be identified by either manual review or NLP techniques. Given the challenges in identifying such responses, these invalid responses were not coded as a new type, with future research potentially exploring how to identify them using other technical means to further improve discrimination methods.

In the field of machine learning, classification task evaluation typically uses metrics such as Accuracy, Precision, Recall, and F1 value (Xu et al., 2024). Using binary classification (positive class, negative class) task confusion matrix elements as examples: (1) True Positive indicates the number of samples that are actually positive class and predicted as positive class by the model; (2) True Negative refers to the number of samples that are actually negative class and predicted as negative class by the model; (3) False Positive is the number of samples that are actually negative class but predicted as positive class by the model; (4) False Negative is the number of samples that are actually positive class but predicted as negative class by the model. Among these, accuracy is the proportion of correctly predicted samples in the total samples, used to measure the overall prediction accuracy of the model. Precision reflects the proportion of samples actually positive among those predicted as positive, in this study embodying the model’s accuracy in predicting invalid responses. Recall measures the proportion of correctly predicted positive samples among actual positive samples, representing the model’s ability to identify invalid responses. The F1 value, as the harmonic mean of precision and recall, is used to balance the relationship between the two.

Based on the performance evaluation paradigm described above, this study precisely defined the prompts for the intelligent agent to identify invalid responses before launching task processing. Two raters coded each response as valid or invalid (with 4 types), counted numbers for each category, and calculated Precision, Recall, and Accuracy values for each. Finally, based on the weighted average of various classification results, the final Precision, Recall, and Accuracy evaluation results were derived (Xu et al., 2024).

## 3 Results

### 3.1 Experiment 1: reliability and validity of SCT items

#### 3.1.1 Content validity

The development of the SCT scale (as described above) was reviewed by 3 experts with experience in SCT application and psychological measurement, ensuring content validity. Of the 1,616 manually scored data points, one expert thoroughly reviewed each item, with a 98.32% approval rate, and minor scoring standard adjustments were made to non-conforming data according to expert opinions.

Upon data review, it was discovered that the item “If I were to score myself” easily triggered defensive responses from participants, with feedback including responses like “you fill it in yourself,” “not good to evaluate,” and “.” Additionally, considering that self-scoring is influenced by multiple complex factors, for single-number responses like “7” or “50,” it is difficult to accurately measure and grasp their specific context. Based on expert opinions, it was decided to remove this item from the study.

#### 3.1.2 Reliability and validity testing

The Cronbach’s *α* of the SCT scale was 0.91, indicating extremely high internal consistency. Using the ICC (2, k) model, the overall test–retest reliability ICC (Intraclass Correlation Coefficient) = 0.93, with a 95% CI of [0.884, 0.968], demonstrating consistency and stability of measurements at different time points.

The Beck Depression Inventory (BDI) was selected as a criterion to test criterion-related validity. BDI, introduced by Beck in 1972 with a 13-item version (*α* = 0.95), uses a 0–3 point scoring system. Although the BDI is a self-report questionnaire, as a classic tool for assessing depression (Jackson-Koku, 2016), if participants can be ensured to respond seriously and objectively, its high correlation with the SCT can indicate that the two are consistent in measuring the construct of depressive symptoms, which provides preliminary evidence for the validity of the projective test (Liu et al., 2014). The SCT total score underwent normality testing, and as the data did not possess normality characteristics, Spearman correlation analysis was employed. Analysis showed that the SCT total score was strongly positively correlated with the BDI total score, with a correlation coefficient *r* = 0.87 (*p* < 0.001), 95% CI [0.80, 0.91]. This validates to some extent the external validity of the SCT scale in assessing depressive symptoms.

### 3.2 Experiment 2: agent scoring performance

#### 3.2.1 Manual scoring correlation analysis

Correlation analysis used correlation coefficients as evaluation criteria. After Spearman correlation calculation, SCT manual scoring and intelligent agent evaluation results showed a strong positive correlation, with a correlation coefficient r = 0.96, 95% CI [0.94, 0.98]. The correlation coefficients for each theme between manual and intelligent agent scoring are shown in Table 2. Researchers have pointed out that a correlation coefficient of 0.7 between machine scoring and manual scoring is sufficient for application in large-scale, high-stakes assessments (Ramineni et al., 2012), and in this study, all theme scores from the intelligent agent met the requirements for large-scale assessment use.

**Table 2:** Final version of 16 SCT items and agent scoring validation.

Item	Human-machine scoring correlation coefficient	Factor loading coefficient			Common factor variance
		Cognitive emotion	Somatic reaction	Interest in things	
My mood is often	0.97***	**0.48**	0.36	0.35	0.49
Facing the future	0.94***	**0.59**	0.37	0.35	0.62
If I make a mistake, I would	0.88***	**0.64**	0.12	0.21	0.47
Looking back, I feel	0.86***	**0.42**	0.34	0.25	0.36
When facing uncertain things,	0.81***	**0.78**	0.11	0.06	0.62
When encountering difficulties, I	0.97***	**0.59**	0.22	0.42	0.59
When I make decisions	0.96***	**0.64**	0.27	0.18	0.52
My sleep situation	0.79***	0.35	**0.69**	0.13	0.62
My appetite	0.90***	0.23	**0.79**	−0.01	0.68
I feel my heartbeat	0.97***	0.26	**0.51**	0.27	0.40
My energy	0.93***	0.53	**0.54**	0.14	0.59
For things I used to like, I	0.90***	−0.27	0.59	**0.49**	0.66
Focusing on tasks for me	0.83***	0.37	0.42	**0.43**	0.51
Participating in social activities for me	0.87***	0.41	0.19	**0.53**	0.49
I think dating	0.93***	0.18	−0.01	**0.79**	0.66
Living in this world	0.82***	0.32	0.32	**0.58**	0.55

*p < 0.05; **p < 0.01; ***p < 0.001. Bold values represent the factors corresponding to the items.

#### 3.2.2 Exploratory factor analysis

Bartlett’s test of sphericity (*χ^2^* = 1567.82, *df* = 120, *p* < 0.001) and Kaiser-Meyer-Olkin (KMO) test (KMO = 0.92) indicated that potential factors might exist between themes, making them suitable for factor analysis. Using principal component analysis and Varimax rotation, three factors with eigenvalues of 1 or higher were identified. Meanwhile, the scree plot showed a sharp drop in the slope for the first three factors, followed by a flattening trend. Based on these two methods, three factors were determined. The cumulative variance explanation rate of the 3 factors was 55.67%, which falls within an acceptable range (Liu et al., 2016; Zhang and Zhou, 2021). Each item had relatively large loading on the corresponding factor [0.43, 0.79]. According to the dimensions measured by each factor, they were divided into cognitive emotion, somatic reaction, and interest in things (Table 2). Although some variance remains unexplained, each item has high loadings on its corresponding factor, indicating that the factor division has clear boundaries. Additionally, both Bartlett’s test of sphericity and the KMO test show that the data is suitable for factor analysis, further supporting the stability of the structure.

#### 3.2.3 Reliability and criterion-related validity

Reliability analysis used Cronbach’s *α* coefficient to measure internal consistency. The overall Cronbach’s α of sample 1 was 0.89, with the three factors of cognitive emotion, somatic reaction, and interest in things showing α values of 0.83, 0.76, and 0.73, respectively. Sample 2 had an overall Cronbach’s α 0.92, with cognitive emotion, somatic reaction, and interest in things showing α values of 0.85, 0.73, and 0.85, respectively.

As the data did not have normal distribution characteristics, Spearman correlation analysis was used. As shown in Table 3, SCT intelligent agent scoring results had significant criterion-related validity with BDI (*N* = 253) and SDS (*N* = 112), with *r*_BDI_ = 0.89, 95% CI [0.86, 0.91]; *r*_SDS_ = 0.85, 95% CI [0.78, 0.90]. Each theme was significantly correlated with criterion total scores and intelligent agent total scores (*p* < 0.001), indicating that SCT intelligent agent scoring has good criterion-related validity (Table 3).

**Table 3:** Criterion-related results.

	BDI total score (*N* = 253)	SDS total score (*N* = 112)	Agent total score (*N* = 112)	Agent total score (*N* = 253)
BDI Total Score	0.89***	0.85***		
Cognitive emotion				
Agent Mood	0.71***	0.66***	0.68***	0.71***
Agent Pessimism	0.74***	0.64***	0.72***	0.78***
Agent Guilt	0.52***	0.55***	0.69***	0.61***
Agent Sense of Failure	0.51***	0.52***	0.59***	0.59***
Agent Excessive Worry	0.53***	0.53***	0.694***	0.616***
Agent Difficulty	0.67***	0.61***	0.73***	0.74***
Agent Decisiveness	0.63***	0.56***	0.65***	0.67***
Somatic reaction				
Agent Sleep	0.60***	0.64***	0.70***	0.64***
Agent Appetite	0.52***	0.45***	0.48***	0.57***
Agent Heartbeat	0.57***	0.43***	0.42***	0.57***
Agent Energy	0.64***	0.63***	0.72***	0.73***
Interest in things				
Agent Concentration	0.61***	0.72***	0.70***	0.69***
Agent Social Interaction	0.59***	0.67***	0.70***	0.68***
Agent Dating	0.39***	0.59***	0.60***	0.52***
Agent Previously Liked	0.37***	0.46***	0.47***	0.40***

*p < 0.05; **p < 0.01; ***p < 0.001.

### 3.3 Experiment 3: invalid response recognition performance

The intelligent agent’s judgment results show that for “Content Omission” type invalid responses, both False Positive and False Negative are 0. This indicates that the model had no misjudgments when identifying “Content Omission” type invalid responses, with accuracy, precision, recall, and F1 values all at 1, demonstrating perfect performance in this category. For “Meaningless” type invalid responses, the intelligent agent’s judgment results also show extremely high accuracy and reliability, with only minimal misjudgment cases. In identifying “Avoidance Interference” and “Off-topic” type invalid responses, although recall rates were slightly lower than other categories, overall performance remained excellent. After calculating weighted averages, the intelligent agent’s overall performance metrics showed a precision of 0.99, indicating that among samples judged as invalid responses by the intelligent agent, the vast majority were indeed invalid; recall was 0.90, meaning the intelligent agent could identify 90% of actual invalid responses; the F1 value was 0.94, indicating good comprehensive performance in invalid response identification tasks (Table 4).

**Table 4:** Comparison of four types of invalid response identification results.

	True positive	True negative	False positive	False negative	Accuracy	Precision	Recall	F1
Content omission	30	930	0	0	1	1	1	1
Avoidance interference	47	899	0	14	0.98	1	0.77	0.87
Meaningless	129	827	1	3	0.99	0.99	0.97	0.98
Off-topic	77	873	0	10	0.98	1	0.88	0.93
Overall					0.99	0.99	0.90	0.94

## 4 Discussion

This study attempted to explore a new paradigm of automated scoring for SCT based on psychodynamic theory. To verify the effectiveness and consistency of its intelligent scoring, we conducted three progressive experiments. Overall, the three experiments in this study maintained high consistency and robustness in results.

### 4.1 Research contributions

First, we proposed a new SCT assessment method based on intelligent agents. Although SCT research has continued for decades (Piotrowski, 2018; Ridgway et al., 2024), related practitioners still primarily use manual scoring methods. Manual scoring is complex and time-consuming, with even experienced raters needing about 20 min to complete one SCT assessment (Hy and Loevinger, 1996), while our developed SCT intelligent agent can complete an assessment in just seconds. The manual scoring process also heavily depends on expert experience, limiting its development and application (Piotrowski, 2018), but whether machine scoring can assist or even replace manual scoring requires particular attention to its validity (Xu et al., 2024). Through experimentation, intelligent agent scoring showed high correlation with manual scoring (*r* = 0.96), and criterion-related validity with BDI and SDS both exceeded 0.8. Additionally, the agent had high filtering efficiency for invalid responses. Therefore, intelligent agent scoring is effective and can achieve human-machine combined scoring or even automated scoring in traditional SCT data analysis.

Second, we developed a localized SCT assessment tool for Chinese college students. SCT items need to be designed according to the characteristics of different cultures and populations (Rogers et al., 2003), and no scholars in China have yet developed depression-themed SCT for college student groups. We verified the good reliability and validity of our SCT items through internal consistency testing, test–retest reliability testing, exploratory factor analysis, external criterion-related validity, and other methods. Additionally, studies typically define six categories of defensive responses in SCT (Loevinger and Wessler, 1970; Ridgway et al., 2024): omission, denial, redundancy, flippancy, comments about the test, and simple association. Combined with the Chinese language context, this study further integrated the six types into four categories: content omission, avoidance interference, meaningless, and off-topic.

Furthermore, the research demonstrates broad application prospects and practical value, primarily reflected in the following aspects: First, exploring the response format of SCT containing projective techniques holds promise for addressing limitations of questionnaire tests in large-scale psychological screening, such as easy faking and social desirability influence (Podsakoff and Organ, 1986). It retains the advantages of projective techniques while utilizing standardized management methods and objective scoring approaches (Sherry et al., 2004), playing a more important role from comprehensive consideration of assessment efficiency and accuracy (Huang, 2016; Tong, 2002). Second, it explores SCT intelligent agent scoring technology, building a complete assessment paradigm, enhancing the explainability of automated scoring processes and technical reproducibility. Third, the assessment results using NLP technology as the core show high accuracy and excellent effects, with the correlation coefficient between machine scoring and manual scoring meeting large-scale, high-stakes assessment standards (Ramineni et al., 2012), proving it a reliable assessment tool. In studies using NLP technology for psychological measurement, Zhang et al. (2024) developed an intelligent agent with human-machine consistency of only 0.71 in psychological trait judgment, while this study achieved a human-machine correlation coefficient of 0.96; Yang et al. (2024) reported an internal consistency Cronbach’s *α* of 0.77 for their intelligent agent measuring depression states, while this study showed *α* = 0.89 for sample 1 and *α* = 0.92 for sample 2. Xu et al. (2024) applied NLP technology for text classification, with text classification accuracy only at 46–55%. In this study, invalid response classification accuracy reached 99%. Fourth, besides psychological health assessment, SCT can also be used in reader attitude evaluation (Lallemand and Mercier, 2022), military task capability judgment (Ridgway et al., 2024), personnel selection screening (Piotrowski, 2018), and other fields, helping to apply SCT intelligent agent scoring in broader testing scenarios.

### 4.2 Limitations and future directions

The study also has some limitations, specifically: First, this research is only the first step in advocating for a paradigm shift in psychological measurement, lacking systematic horizontal comparative studies to ensure its reliability and applicability in psychological screening or diagnostic processes, limiting judgment of paradigm superiority. Moreover, projective tests can to some extent prevent participants from falsifying their responses, but cannot completely avoid it (Sherry et al., 2004). This is specifically manifested in the discovery, in experiments on detecting invalid responses, of a type of invalid response that cannot be detected by either manual review or NLP techniques.

Second, the participants in this study were recruited through sample service platforms, and the randomness of responses in scenarios without answer rewards was not considered, so they may not represent the broader college student population. Moreover, the applicability of the study to other groups remains uncertain.

Meanwhile, there are prominent ethical challenges: To begin with, psychological assessments involve a large amount of personal sensitive information (such as depression status and inner thoughts), leading to the risk of privacy leakage during data collection, storage, and use. Besides, the training data of large language models (LLMs) may contain implicit group biases, which could result in algorithmic bias (Markowitz, 2024) and affect the fairness of assessment results for college students from different backgrounds (e.g., gender, region, and family environment). Third, the risk of misprediction cannot be ignored—misclassifying non-depressed individuals as depressed may trigger unnecessary psychological anxiety, while failing to identify truly depressed individuals may delay intervention opportunities.

Finally, to ensure the safe use of LLMs, regular monitoring and maintenance of the agent’s operating status are required, and it must be used under the supervision of qualified mental health professionals (Zhang et al., 2024), which imposes certain cost constraints on its application in resource-poor areas.

To address these limitations, future research will conduct in-depth comparisons between SCT intelligent agents and questionnaire scales, other NLP-based assessment methods, analyzing differences in screening accuracy (like sensitivity, specificity or AUC), efficiency, and user acceptance, and analyzing the intelligent agent’s ability to identify depression of different level, clarifying its application boundaries. Furthermore, we will consider incorporating a covert validity scale, such as through methods like posing questions about similar scenarios in different phrasings and checking for consistency in responses, to prevent participants from falsifying.

For sample selection, experiments will be conducted through schools, mental health centers, and other channels in non-reward scenarios (such as classroom assignments, visitor surveys) to observe changes in answer quality and evaluate the intelligent agent’s anti-interference ability in real scenarios. Meanwhile, future analysis will include more diverse participants with more varied clinical backgrounds, so as to make the research findings more widely applicable.

In terms of ethical safeguards, full-life-cycle data management will be strengthened, with encryption and anonymization technologies used to protect privacy. Efforts will be made to reduce biases by incorporating diversified training data and regularly auditing algorithmic fairness. The model will be directly fine-tuned, or early warning mechanisms for misprediction and rapid error correction processes will be established, while clarifying the supervisory responsibilities of mental health professionals (e.g., real-time review of high-risk results).

Furthermore, attention will be paid to models with advanced algorithms and low-cost computing power for agent design. Automated monitoring systems will be developed to ensure that the output of agents complies with ethical norms, and emergency intervention interfaces will be set up to promote the practical application of SCT agents.

Looking ahead, SCT is expected to integrate into multi-modal assessment systems, combining with voice recognition, facial expression feature analysis, and other technologies. Multi-modal approaches focus on extracting and fusing semantic information from text, image, and audio data (Wu et al., 2024), and intelligent agents may be able to simultaneously analyze participants’ facial expression features, voice emotion features, and language text content. At present, multi-modal fusion solutions have shown initial success in related research fields (Yin et al., 2024), and it can be expected that LLMs will achieve even more remarkable progress in the near future.

## Data Availability

The original contributions presented in the study are included in the article/Supplementary material, further inquiries can be directed to the corresponding authors.
